# Supplementary material for: A method to improve the reproducibility of findings from epigenome- and transcriptome-wide association studies
Source: bioRxiv. 2023 Mar 31:2023.03.29.534761. Preprint. [Version 1] doi: 10.1101/2023.03.29.534761 (PMC10081238; doi:10.1101/2023.03.29.534761)
Supplement: Supplement 1 [file media-1.docx]

Supplemental figures for the article: **A method to improve the reproducibility of findings from epigenome- and transcriptome-wide association studies.**

Figure S1. QQ plot regular MWAS

Figure S2. QQ plot for robust MWAS
